# Genome-Wide Detection of Copy Number Variations Associated with Miniature Features in Horses

**DOI:** 10.3390/genes14101934

**Published:** 2023-10-13

**Authors:** Md. Panir Choudhury, Zihao Wang, Min Zhu, Shaohua Teng, Jing Yan, Shuwei Cao, Guoqiang Yi, Yuwen Liu, Yuying Liao, Zhonglin Tang

**Affiliations:** 1 Kunpeng Institute of Modern Agriculture at Foshan, Agricultural Genomics Institute at Shenzhen, Chinese Academy of Agricultural Sciences, Foshan 518124, China; cpanir@yahoo.com (M.P.C.); yiguoqiang@caas.cn (G.Y.); liuyuwen@caas.cn (Y.L.); 2 Shenzhen Branch, Guangdong Laboratory of Lingnan Modern Agriculture, Key Laboratory of Livestock and Poultry Multi-omics of MARA, Kunpeng Institute of Modern Agriculture at Foshan, Agricultural Genomics Institute at Shenzhen, Chinese Academy of Agricultural Sciences, Shenzhen 518124, China; 3 Bangladesh Livestock Research Institute, Ministry of Fisheries and Livestock, Savar, Dhaka 1341, Bangladesh; 4 Animal Husbandry Research Institute, Guangxi Vocational University of Agriculture, Nanning 530002,China; wangzh761006@163.com (Z.W.); zjqwtm543@foxmail.com (M.Z.); tsh088@126.com (S.T.); gxxmyjsrs@126.com (J.Y.); cao_shuwei@126.com (S.C.); 5 Guangxi Veterinary Research Institute, Nanning 530001, China

**Keywords:** horse, copy number variations, whole-genome resequencing, genome-wide detection

## Abstract

Copy number variations (CNVs) are crucial structural genomic variants affecting complex traits in humans and livestock animals. The current study was designed to conduct a comprehensive comparative copy number variation analysis among three breeds, Debao (DB), Baise (BS), and Warmblood (WB), with a specific focus on identifying genomic regions associated with miniature features in horses. Using whole-genome next-generation resequencing data, we identified 18,974 CNVs across 31 autosomes. Among the breeds, we found 4279 breed-specific CNV regions (CNVRs). Baise, Debao, and Warmblood displayed 2978, 986, and 895 distinct CNVRs, respectively, with 202 CNVRs shared across all three breeds. After removing duplicates, we obtained 1545 CNVRs from 26 horse genomes. Functional annotation reveals enrichment in biological functions, including antigen processing, cell metabolism, olfactory conduction, and nervous system development. Debao horses have 970 genes overlapping with CNVRs, possibly causing their small size and mountainous adaptations. We also found that the genes GHR, *SOX9*, and *SOX11* may be responsible for the miniature features of the Debao horse by analyzing their overlapping CNVRs. Overall, this study offers valuable insights into the widespread presence of CNVs in the horse genome. The findings contribute to mapping horse CNVs and advance research on unique miniature traits observed in the Debao horse.

## 1. Introduction

Copy number variations (CNVs) are a type of change in the structure of the genome in which segments of DNA sequence variants spanning from 50 bp to several Mb are widely distributed throughout the genome. Compared to the reference genome, CNVs have large-scale insertions, deletions, duplications, inversions, and translocations [1,2,3,4]. CNVs are common in both human and animal genomes. CNVs are a significant source of genomic diversity and have been related to phenotypic variation, environmental adaptability, and disease susceptibility [5,6,7,8] in humans and livestock [9]. CNVs play a role in the adaptation of horse populations to different environments, distinctive phenotype features, population diversity, and evolution. Specific CNVs influence susceptibility or resistance to certain equine diseases by affecting the function or expression of associated genes. CNVs also impact the genes that are crucial for the interaction between the horse and its microbiome, influencing digestion, nutrient absorption, or susceptibility to infections [3,10,11,12,13]. Previous genome-wide association investigations have found a significant relationship between copy-number variants and the development of short stature in horses [14,15,16,17]. Despite this, much remains to be learned about the comprehensive copy number differences in the genome-wide mapping of horses, specifically focusing on identifying genomic regions associated with miniature features in horses. To reach this goal, data from whole-genome resequencing was used to make a horse genome-wide CNV map.

The efficiency and precision of CNV identification at the whole-genome level have greatly improved because of recently developed sequencing technologies like next-generation sequencing (NGS) [18]. Copy number variation could considerably impact an individual’s phenotype [19]. CNVs contribute to the diversity of genomics by assessing animal breeds and strengthening the precision of genomic models of evaluation [20]. Recent advances in sequencing technology research have made it easier for researchers to identify CNVs throughout the sheep [4,21,22,23,24,25], goats [26,27], cattle [28,29,30], pigs [5,31,32], horses [33,34], chickens [35,36], dogs [37,38], and rabbits genomes [39].

Over 45–55 million years ago, horses (*Equus caballus*), a member of the Equidae family, developed from a small multi-toed mammal to a substantial single-toed mammal. It is a biological model for studying exercise physiology, adaptive responses, and human disorders like infertility and inflammatory diseases [40,41]. China has the world’s largest population of horses, primarily found in rural areas, but their numbers are rapidly decreasing. Horses have enormously impacted China’s political, economic, and military history [42]. The Debao horse is a Chinese horse breed with a miniature body size. Debao horses have unusual morphoanatomical adaptations to aid in hilly activities [43,44]. The Baise horse, which is also a mountain breed from China’s Guangxi Zhuang Autonomous Region, is known for its muscular body, strong legs, and unique head profile, making it versatile in transportation, agriculture, and riding [3]. On the other hand, Warmblood horses, known for their calm temperament and agility, excel in equestrian sports like dressage, show jumping, and eventing. Warmblood horses were introduced to China from Germany [42].

Systematic human selective breeding of horses has emphasized conformational and performance traits, creating highly specialized breeds that are frequently identifiable by the highest possible height at the withers [45,46]. Horses exhibit significant body size and wither height diversity due to extensive breeding and artificially selected breeding throughout domestication. Body size is currently one of the essential considerations in evaluating and classifying various breeds and a necessary component in breeding initiatives to improve marketability and performance [17,44,47]. Body size is also one of the most significant quantitative traits under evolutionary investigation and varies significantly between breeds and populations within species [34]. Presently, molecular components associated with body size have been studied in farm animals and humans. Human body height was found to be associated with two chromosomal regions around the genes *LCORL/NCAPG* and *ZFAT* [14]. Previous research has identified a small number of loci for the genetic basis of size variation in Western ponies and horses [12,34,48,49,50]. The genes *MRas* and *ATF3* in the *GHR* shed light on the genetics of the Debao pony of small stature. To understand the genetic basis of short size, genes such as *ECM*, *ALPL*, *IHH*, *MMP23*, *TIMP4*, *COL10A1*, *Sox9*, *Sox6*, *Sox8*, *Sox11*, *COL2A1*, and *COL9A1* have been identified in the juvenile stage of the Debao horse [51]. A recent study showed that the genes *TBX3*, *TBX5*, and *HMGA2* play a role in the genetic underpinnings of Chinese pony dwarfism [52].

The purpose of this study was to use whole-genome resequencing data to establish horse genome-wide CNV mapping. Furthermore, we concentrated on the Debao horse population to identify candidate genes associated with miniature features. Our study advances the Debao horse’s breeding and conservation efforts by presenting new data on the genome-wide CNVs of horses.

## 2. Materials and Methods

### 2.1. Sample and Sequencing Information

The horse breeds studied were Debao (DB) horses, Baise (BS) horses, and Warmblood horses from the Animal Husbandry Research Institute in Guangxi Zhuang Autonomous Region, China. Twenty-six blood samples, ten samples from Debao, ten samples from Baise, and six samples from Warmblood horses were extracted using the DNeasy Blood & Tissue Kit (Qiagen, Beijing, China), and DNA integrity was assessed using aga-rose gels for whole-genome resequencing. PE sequencing libraries were created and then sequenced on the Illumina NovaSeq 6000 platform, producing 150-bp reads with an in-tended depth of 10× coverage. All resequencing data were from our previous study [53].

### 2.2. Data Processing

The data processing pipeline was detailed in our previous study [53]. Briefly, the FastQC tool v0. 12.1 (http://www.bioinformatics.babraham.ac.uk/projects/fastqc/, viewed on 4 April 2023) with the default settings were used to assess the quality of our data. We applied BWA [54] with the parameters ‘mem 4 -k 32 -M’ to map each sample’s clean reads against our newly reconstructed horse genomic data. SAMtools v1.18 was used to convert alignment files to BAM files [55]. Picard3.0. (http://broadinstitute.github.io/picard/, accessed on 4 April 2023) is also utilized to filter and eliminate duplicates from the aligned BAM data. The resulting sequencing quality was sufficient for further analysis.

### 2.3. Screening of the Initial Set of CNVs

Genome-wide copy number variation analysis on horse next-generation sequencing data was carried out using CNVcaller [56]. To extract CNVs, the data were separated into three groups based on the three horses: Debao (DB), Baise (BS), and Warmblood (WB). Since the quality of the reference genome significantly influences the detection of CNV, in this study, we used the assembled Debao horse genome from our previous study [53] as reference genomes. It divided the reference genome according to the window size of 800 bp to construct the reference genome database. The number of reads in the window and the reads on the alignment corrected the copy number, corrected and standardized the GC content, and finally, the genotype was determined. All other statistical analysis was calculated using the R 4.2.2 package. 

### 2.4. Merge CNVR Collections

Based on the screened CNV data information, the boundaries of CNVRs are initially judged according to the distribution of absolute copy numbers, the frequency of mutations, and the significant correlation of adjacent windows, and finally, the adjacent CNVRs with significant correlations in the distribution of copy numbers in the population were merged using the merge function in BEDTools v2.25 [57] to obtain the final copy number variation detection result. In addition, this study also filters the CNVR data set according to the Silhouette score and chromosome. The silhouette score was calculated based on the silhouette coefficient using the R 4.2.2 package. The silhouette coefficient is an evaluation method for the clustering effect. In this study, according to the silhouette coefficient greater than 0.6, the CNVR set was screened, and the CNVR located in the sex chromosome and Scaffold region was removed.

### 2.5. CNVR Annotation and Functional Enrichment Analysis

Based on our reference genome of the Debao horse, the NCBI Genome Database was used to obtain the gene content of the identified CNVRs. The Ensemble database was utilized to identify the genes involved within or partly overlapping with the horse CNVRs using ANNOVAR software [58]. Functional enrichment analysis of the CNVR-harboring genes was performed using human orthologs to better understand these CNVRs’ functions. To determine their functional enrichment, we used the DAVID program (version 2023q1) [59] to conduct Gene Ontology (GO) categorization [60] and Kyoto Encyclopedia of Genes and Genomes (KEGG) pathway annotation [61] on these CNVs genes.

## 3. Results

### 3.1. Genome-Wide Identification of CNV in Three Horse Breeds

Based on our previous study [53], ten Debao horses, ten Baise horses, and six Warmbloods resequencing data showed an average depth of around 10.87× (Table S1), which were used to detect CNV in horses at the genome-wide level. After filtering, 26 samples of three horse breeds generated 18,974 CNVs; the figures in parenthesis represent the CNV counts of a particular horse breed (Table 1). The DB horses had the most (6564) CNVs, including 4028 breed-specific CNVs, whereas the WB horses had the lowest number. CNVs ranged in length from 1599 to 891,199 bp, with an average length of 5364.85 bp per breed. The maximum length of CNV in DB horses was 891,199 bp, and the average CNV in WB horses was 5598.86 bp. The Warmblood horse population has the highest average number of individual CNVs detected (984.83) and unique breed-specific CNVs (613.33).

### 3.2. Diversity Analysis of CNVRs in Different Breeds

**Figure 1:**
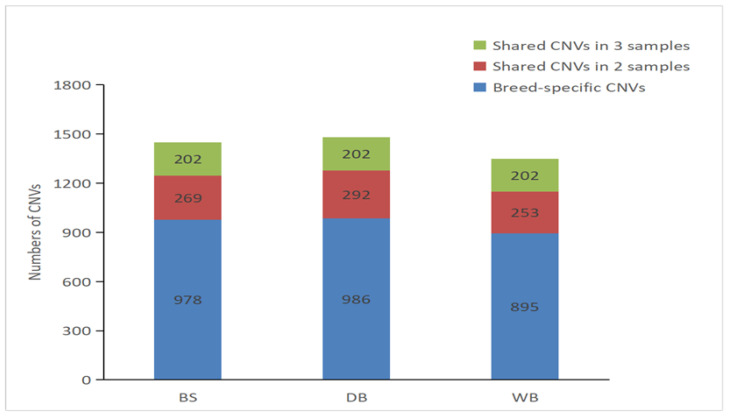
The distribution of CNVs in various horse breeds. Breed-specific and shared CNV distribution throughout horse breeds. Breed-specific CNVs are shown in blue. Green denotes CNVs shared by three breeds, whereas red denotes CNVs shared by two breeds.

After assembling overlapping CNVs, 4279 CNVRs were identified in 31 pairs of autosomes from the three horse breeds (Figure 1). Among them, the DB horse population shows the largest number of CNVRs (1480), while the WB breed presents the least (1350) (Table 2) due to the intensely artificial selection. The figures in brackets represent breed-specific CNVR counts. We defined a breed-specific CNVR as any CNVR found exclusively in one breed and not shared by any other breed.

**Figure 2:**
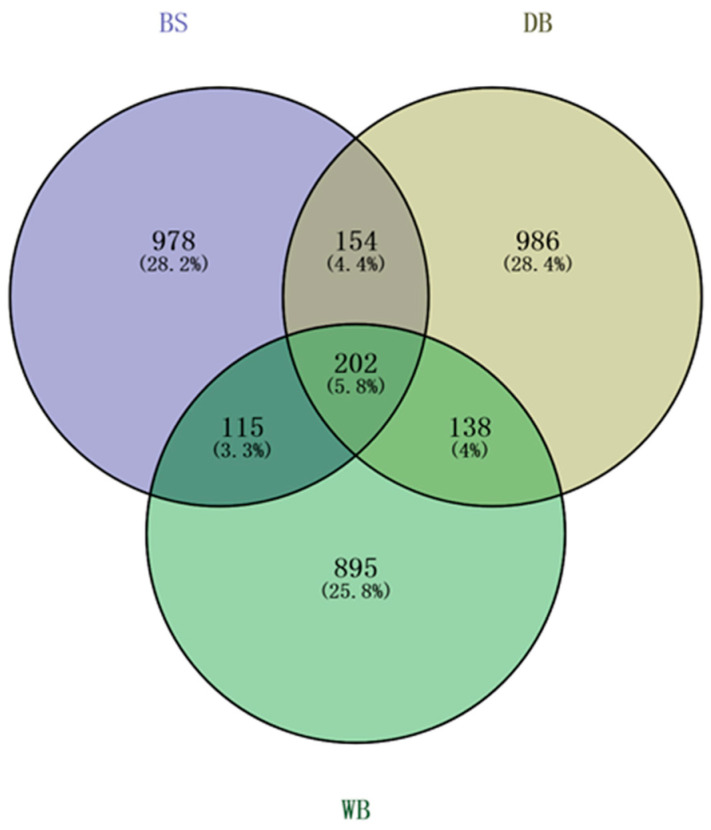
The number of CNVRs here identified and shared among the three horse breeds.

Genome-wide CNVRs from the three populations were summarized in this study. BS, DB, and WB had 978, 986, and 895 unique CNVRs, respectively, whereas 202 CNVRs were found in all three horse breeds (Figure 2).

### 3.3. Merge and Functional Analysis of CNVRs from Different Horse Breeds

**Figure 3:**
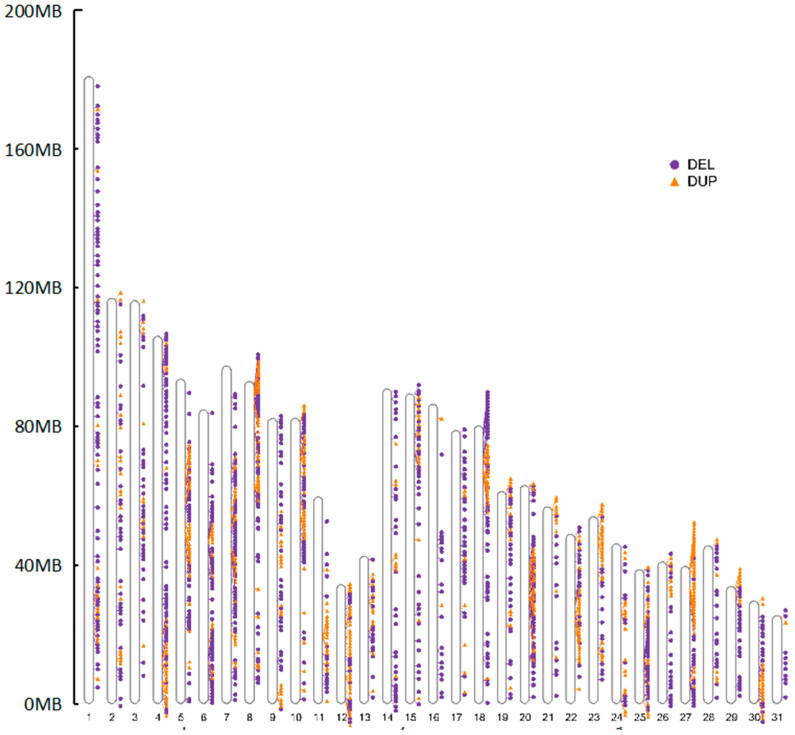
Map of CNVRs in the horse genome. Circles and triangles represents DEL and DUP, respectively.

**Figure 4:**
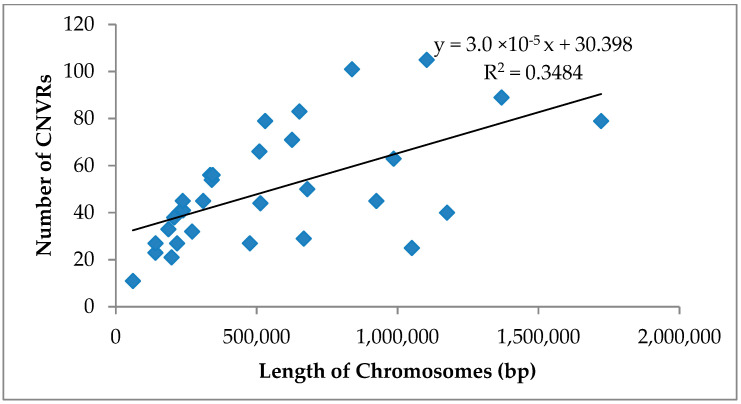
Correlation between chromosomal length and the number of CNVRs.

Since different breeds of horses have the same CNVR, a complete map of the CNVR and a comparative overview of their genomes were created by removing the repeated CNV regions between breeds (Table 3 and Figure 3). After the deletion of duplicate CNVRs, 1545 CNVRs were generated from the genomes of 26 horses. The lengths of the identified CNVRs varied significantly, with an average length of 11,178.55 bp. The number of CNVRs on chromosome 20 is the highest among the 31 autosomes, whereas the number on chromosome 31 is the lowest (Figure 4). The CNVR scales on each chromosome were significantly varied. Chromosomes 6, 7, 12, 13, 20, 22, 23, 28, and 29 contained dense CNVRs that covered more than 1% of genomic sequences, with chromosome 12’s CNVRs encompassing 2.62% of the chromosome’s sequences, while chromosome 31 covered only 0.23% (Table 3).

There was no correlation between the incidence rate of CNVRs and chromosomal length (Figure 4) since the longest chromosome 1 had only 89 CNVRs, while chromosome 20 (approximately one-third the length of chromosome 1) had 105 CNVRs. The findings of this study coincide with those of prior studies [13].

**Figure 5:**
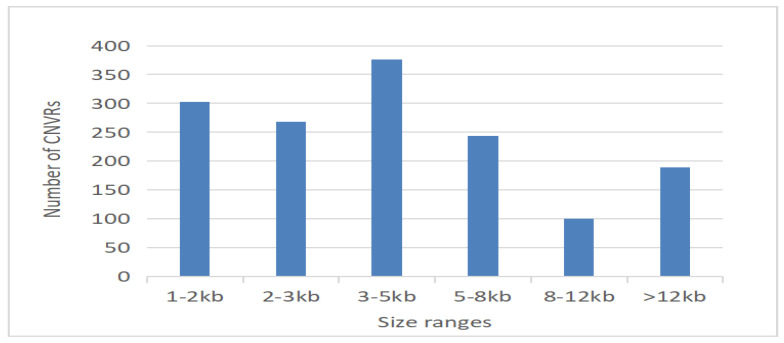
Size range distribution of the CNVRs detected.

Statistics of CNVRs on equine autosomes. The length of CNVRs has been classified into six regions: 1–2 Kb, 2–3 Kb, 3–5 Kb, 5–8 Kb, 8–12 Kb, and >12 Kb (Figure 5). The greater the magnitude of the copy number variation (CNV), the higher the level of ease in its detection.

### 3.4. Functional Annotation of CNVR in Horse

**Figure 6:**
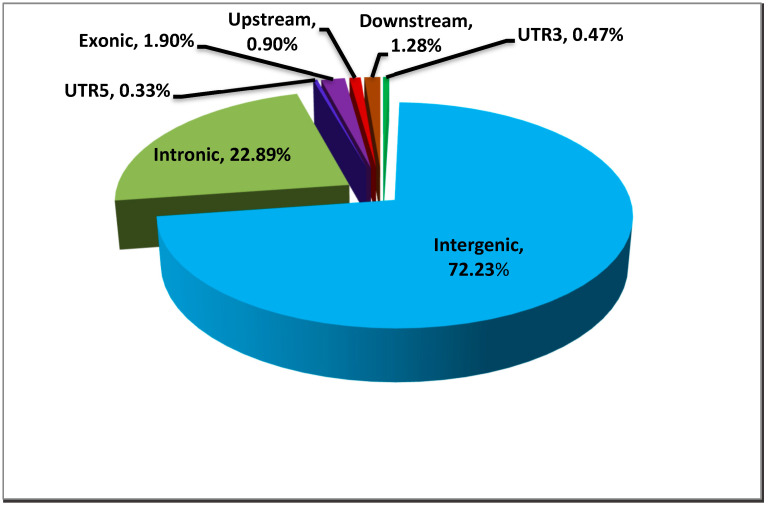
Functional classifications of the detected CNVRs.

We performed functional analysis and grouped these CNV-affected genes to determine how CNVs might affect gene biotypes in horses. The functional annotation of 1545 CNVRs from 26 samples showed that 72.23% of CNVRs were in the intergenic region of the genome, 22.89% were in the intron region of the genome, and 1.90% were in the exon region of the genome (Figure 6). Molecular function is mainly related to neurotransmitter receptor activity, calcium ion binding, amino acid activity, protein kinase activity, and the heme binding site. Regarding cellular components, these genes were highly enriched in the cytoplasm, plasma membrane, synapses, and cell surface. Pathway analyses in the Kyoto Encyclopaedia of Genes and Genomes (KEGG) showed that it annotated many genes with known highly variable copy numbers that significantly enriched antigen processing and presentation, functions such as cell metabolism, olfactory conduction, and nervous system development (the corrected *p*-value is less than 0.05 and the number of genes is greater than or equal to 10) (Table 4).

Similarly, We used Gene Ontology (GO) analysis to assess the possible biological implications of these CNV genes. GO enrichment was shown to be significant in three areas: biological process, cellular component, and molecular function (the corrected *p*-value less than 0.05, number of genes more than or equal to 10) (Table 5). Antigen processing and presentation, chemical synaptic transmission, cell morphogenesis, negative regulation of apoptotic processes, intracellular message transmission, and homophilic cell adhesion via plasma membrane adhesion molecules are representative biological processes. Previous studies supported our results [3,5].

**Figure 7:**
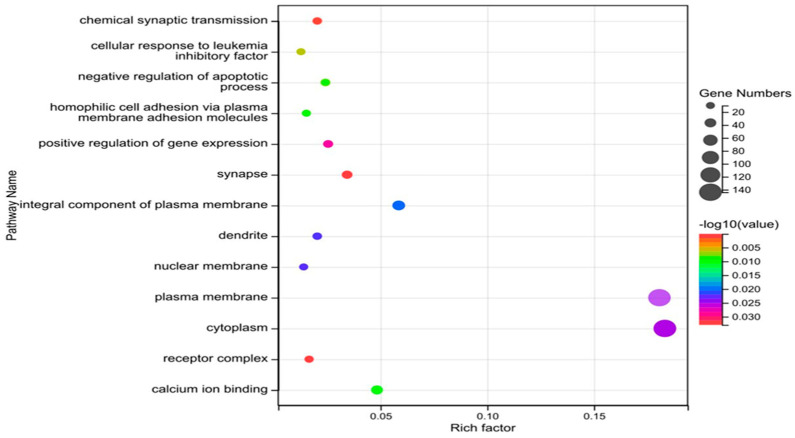
Statistics of GO pathways.

The pathways are illustrated with a bubble diagram. The names of the pathways are given in the legend on the left. The abscissa displays the enrichment factor, which is the ratio of the percentage of genes identified to a pathway in a particular gene to the overall balance of genes designated to that route. The higher the enrichment factor, the more the differentially expressed genes in this pathway are enriched. The size and color of the dots denote the number of enriched genes and the level of significance, respectively (Figure 7).

In this study, 1089 genes (excluding LOC genes) were annotated in the CNVR data set in 26 samples of three horse breeds (Tables S2 and S3), and 970 genes (excluding LOC genes) were annotated in the CNVR data set in 10 Debao horse samples (Tables S4 and S5). Many genes with known highly variable copy numbers were annotated based on the EquCab 3.0 genome. These genes were significantly enriched in antigen processing and presentation, cell metabolism, olfactory conduction, nervous system development, and other functions (the corrected *p* value is less than 0.05, and the number of genes is greater than or equal to 10).

Through GO analysis of these CNV genes, we found that the following genes (*CYP2C108*, *PLA2G4A*, *ITGA8*, *GHR*, *CYP11B1*, *CYP2B46*, *BCL2A1*, *RBFOX*, *PLAAT1*, *CAPN9*, *SULF2*, *CAPN2*, *ADAMTS15*, *NOX3*, and *PCDH18*) were associated with the body structure in all horse breeds. Additionally, a distinct set of genes (*GRID2*, *SOX9*, *CYP2B6*, *FBP1*, *FBP2*, *MAPKAP1*, *STX17*, *TBCA*, and *SOX11*) were found to be associated with body structure in the Debao horse breed. We analyzed our data using KEGG Pathway Database annotations. We found that eleven genes were associated with the horse body structure: six of them (*CYP2C108*, *PLA2G4A*, *ITGA8*, *GRID2*, *CYP11B1*, and *CYP2B46*) were related to the three breeds here analyzed, while five genes (*GHR*, *SOX9*, *CYP2B6*, *FBP1*, and *FBP2*) only to the Debao horse. Based on gene ontology and genetic pathway research, we hypothesize that the *GHR* and *ADAMTS15* genes in CNVs have some relationship with miniature features in horses. *GHR*, *SOX9*, and *SOX11* genes are shown to overlap with CNVRs and play essential regulatory functions in the pathway.

## 4. Discussion

A significant amount of study has been undertaken in recent years on the genetic variants underlying the genetic diversity of horse breeds. Furthermore, the prevalence of CNVs in horses and their effect on phenotypic variation remain understudied. In the current study, we studied copy number variations among horse breeds from China using data from whole-genome resequencing. The recent investigation revealed more CNVs than previous studies [3,13,17,34,62]. This could be due to different experimental sample sizes or CNV detection technologies.

This study’s findings on CNV length ranges align with previous research [3]. We found that each horse shared a significant percentage of its CNVs with at least one other horse, which is consistent with their results. Our results provide relevant insights that complement the study of [13]. In this study, we identified 202 unique CNVs across all horse breeds, all of which were breed-specific. Additionally, we observed the presence of CNVs ranging from 253 to 986, which two or more horse breeds shared. The recent study not only covered a significant number of samples, but it also identified CNV fragments comprising a higher percentage of the genome, resulting in more CNV variation as well as understanding than earlier studies on CNVs in horse breeds in China. This may be due to the diverse research platforms, experimental sample sizes, and detection methodologies of CNVRs [3,5,43].

The distribution of CNVRs in this study is consistent with that discovered in other studies. Chromosome 20 had the most significant number of CNVRs, while Chromosome 12 had the highest proportion of its genome covered by CNVRs. According to an earlier study, the percentage of CNVRs is higher in shorter lengths, while the ratio is lowest in long-length CNVRs [3,13,43,62,63]. These findings suggest that the proportion of CNVR differs across breeds, likely attributable to the genetic origins of these populations or the varied samples gathered for each breed. This observation may be addressed by the higher frequency of shorter CNVRs in the equine genome [3,64,65].

Many exhaustive CNV studies regarding horses have been undertaken, focusing mainly on CNV discovery and reporting their numbers and categories. In contrast, just a few studies looked at the possible relationship between CNVs and complex features. Several studies have shown that genes that overlap with CNVs influence the characteristics of domestic animals. For example, some coat colors in horses, pigs, and sheep are controlled by genes that are impacted by CNVs [3,66]. Further research has revealed that CNV-affected genes also contribute to adaptations in horses [3,13], and some are found to influence growth and body size in horses [17,33,49]. Studies on cattle have shown a relationship between growth features such as cannon circumference, body slanting length, chest girth, and body weight and the loss and normal CNV types of the *CLCN2* gene [67]. Copy number variant regions (CNVRs) were explored in order to better understand the genes linked with adaptability (BLA-DQB, EGLN2) and coat color (KIT, MITF) in cattle [2].

Surprisingly, some CNVRs were regarded as breed-specific or were identified in a few species, indicating that the variability may contribute to determining phenotypic characteristics and differentiating between variety-specific traits [68]. In the present study, breed-specific CNVRs were identified, and the most significant number of breed-specific CNVRs was detected in the Debao horse. *GHR* expression was comparatively low in the long bones of the Debao horse. By downregulating the *MAPK* signaling cascade, *GHR* may reduce Debao horse height. The candidate genes *Sox9* and *Sox11* were found to promote the early occurrence of bio mineralization and epiphyseal closure in Debao ponies [51]. The candidate gene *ADAMTS15* is associated with muscle development, such as myoblast fusion in thoroughbred horses [69]. *ADAMTS* have been reported to play an essential role in damaged cartilage tissues and the musculoskeletal system and restrict locomotor functions in horses [70].

Based on the overlap of CNVRs, we found that the genes *GHR*, *SOX9*, and *SOX11* may have miniature features in the Debao horse. We also assume that the *GHR* and *ADAMTS15* genes in CNVs are related to small body size-related traits in the studied horse breed. They were also screened out based on biofunction, and they may have been identified as candidate genes for the miniature features in the Debao horse.

## 5. Conclusions

In this study, whole-genome resequencing data at the level of the genomic structure were used to make a complete and detailed CNV-based genome-wide model map of horses. Numerous distinct and breed-specific CNVRs in horse breeds support the idea that CNVs are common in the horse genome. Our analysis of the overlapping CNVRs of genes *GHR*, *SOX9*, and *SOX11* has revealed that these may be responsible for the Debao horse’s miniature features. Furthermore, research is necessary to determine the roles of these types of genes in horses. These findings contribute to our understanding of CNVs in the horse genome and have potential implications for future research, providing new insights into the molecular mechanisms underlying miniature features in horses.

## Figures and Tables

**Table 1:** The statistical results on CNV detection.

Breed	Sample Number	Number of CNVs (Breed Specific CNVs)	Average Number of CNVs Per Individual	Average CNV Length (bp)	Range of CNVS Lengths (bp)
BS	10	6501 (3981)	650.10 (398.10)	5295.85	1599–485,599
DB	10	6564 (4028)	656.40 (402.80)	5199.85	1599–891,199
WB	6	5909 (3680)	984.83 (613.33)	5598.86	1599–453,599
Total	26	18,974 (11,689)	729.77 (449.58)	5364.85	1599–891,199

**Table 2:** Statistical values from CNVR identification.

Breed	Sample Number	CNVRs	Duplications	Deletions	Average Number of CNVRs Per Individual
BS	10	1449 (978)	554 (308)	895 (670)	144.90 (97.80)
DB	10	1480 (986)	572 (305)	908 (681)	148.00 (98.60)
WB	6	1350 (895)	516 (285)	834 (610)	225.00 (149.17)
Total	26	4279 (2859)	1642 (898)	2637 (1961)	164.58 (109.96)

**Table 3:** The descriptive statistics of the CNVRs on the autosomes of horses.

Chr	Chromosomes Length (Mb)	Number of CNVRs	Length of CNVRs (bp)	Percentage (%)	Average Length of CNVRs (bp)
chr_1	177.90	89	1,368,311	0.73%	15,374.28
chr_2	114.97	56	334,744	0.28%	5977.57
chr_3	114.24	45	309,955	0.26%	6887.89
chr_4	104.15	83	651,517	0.60%	7849.60
chr_5	92.01	71	625,129	0.65%	8804.63
chr_6	83.24	63	985,937	1.13%	15,649.79
chr_7	95.73	79	1,721,921	1.72%	21,796.47
chr_8	91.31	101	838,299	0.88%	8299.99
chr_9	80.83	54	340,746	0.40%	6310.11
chr_10	80.93	66	509,534	0.60%	7720.21
chr_11	58.56	33	186,767	0.30%	5659.61
chr_12	33.69	45	924,355	2.62%	20,541.22
chr_13	41.78	27	475,973	1.09%	17,628.63
chr_14	89.19	40	223,160	0.24%	5579.00
chr_15	87.75	50	679,150	0.74%	13,583.00
chr_16	84.85	21	198,379	0.22%	9446.62
chr_17	77.42	38	207,162	0.26%	5451.63
chr_18	78.73	79	529,921	0.64%	6707.86
chr_19	60.06	41	238,759	0.38%	5823.39
chr_20	61.77	105	1,102,695	1.70%	10,501.86
chr_21	55.74	23	140,777	0.24%	6120.74
chr_22	48.01	44	513,156	1.02%	11,662.64
chr_23	52.94	40	1,175,160	2.12%	29,379.00
chr_24	45.28	27	217,973	0.46%	8073.07
chr_25	37.98	45	237,555	0.60%	5279.00
chr_26	40.14	27	141,173	0.34%	5228.63
chr_27	38.91	56	343,544	0.84%	6134.71
chr_28	44.74	25	1,050,375	2.24%	42,015.00
chr_29	33.23	29	666,771	1.91%	22,992.10
chr_30	29.06	32	271,168	0.89%	8474.00
chr_31	24.94	11	60,789	0.23%	5526.27
Total	2160.09	1545	17,270,855	0.76%	11,178.55

**Table 4:** Analysis of KEGG pathways.

KEGG Pathway	Gene Count	*p*-Value
ecb04612:Antigen processing and presentation	14	1.41 × 10^−4^
ecb05321:Inflammatory bowel disease	12	3.89 × 10^−4^
ecb04726:Serotonergic synapse	16	4.14 × 10^−4^
ecb05145:Toxoplasmosis	15	6.58 × 10^−4^
ecb05150:Staphylococcus aureus infection	14	0.001589502
ecb05323:Rheumatoid arthritis	13	0.001979771
ecb04514:Cell adhesion molecules	18	0.002371425
ecb05332:Graft-versus-host disease	11	0.002491976
ecb05140:Leishmaniasis	11	0.004276101
ecb05330:Allograft rejection	10	0.004827152
ecb04940:Type I diabetes mellitus	10	0.010278294
ecb05416:Viral myocarditis	10	0.010278294
ecb04080:Neuroactive ligand-receptor interaction	28	0.010966373
ecb04742:Taste transduction	10	0.014830627
ecb05320:Autoimmune thyroid disease	10	0.017015378
ecb04921:Oxytocin signaling pathway	14	0.01768501
ecb04640:Hematopoietic cell lineage	12	0.019640595
ecb05166:Human T-cell leukemia virus 1 infection	19	0.026746256
ecb04658:Th1 and Th2 cell differentiation	10	0.028135098
ecb05152:Tuberculosis	16	0.028156201
ecb04530:Tight junction	15	0.03200504
ecb04024:cAMP signaling pathway	17	0.044243718
ecb01100:Metabolic pathways	86	0.048244214

**Table 5:** Analysis of GO pathway.

Category	Term	Gene Count	*p*-Value
GOTERM_BP_DIRECT	GO:0019882~antigen processing and presentation	10	2.39 × 10^−5^
GOTERM_BP_DIRECT	GO:0007268~chemical synaptic transmission	15	0.00368292
GOTERM_BP_DIRECT	GO:0000902~cell morphogenesis	10	0.005961947
GOTERM_BP_DIRECT	GO:0043066~negative regulation of apoptotic process	19	0.024972288
GOTERM_BP_DIRECT	GO:0035556~intracellular signal transduction	24	0.041473254
GOTERM_BP_DIRECT	GO:0007156~homophilic cell adhesion via plasma membrane adhesion molecules	11	0.046559993
GOTERM_CC_DIRECT	GO:0005887~integral component of plasma membrane	55	0.00307342
GOTERM_CC_DIRECT	GO:0045202~synapse	23	0.009198062
GOTERM_CC_DIRECT	GO:0005737~cytoplasm	159	0.030272449
GOTERM_CC_DIRECT	GO:0009986~cell surface	24	0.041485424
GOTERM_MF_DIRECT	GO:0030594~neurotransmitter receptor activity	10	0.007542982
GOTERM_MF_DIRECT	GO:0005509~calcium ion binding	41	0.011430849
GOTERM_MF_DIRECT	GO:0004712~protein serine/threonine/tyrosine kinase activity	19	0.017675014
GOTERM_MF_DIRECT	GO:0004672~protein kinase activity	15	0.045718707
GOTERM_MF_DIRECT	GO:0020037~heme binding	13	0.047036578
GOTERM_MF_DIRECT	GO:0004674~protein serine/threonine kinase activity	21	0.047891973

## Data Availability

The sequencing data were from our previous study, and have been deposited in the NCBI database under the BioProject accession PRJNA1005486. All relevant data are available from the corresponding author upon reasonable request.

## Supplementary Materials

The following supporting information can be downloaded at: https://www.mdpi.com/article/10.3390/genes14101934/s1; The following files, namely: Supporting File S1: CNVs and CNVRs identified in this study; Table S1. Summary of samples sequenced in study; Tables S2 and S3: KEGG pathway analysis and GO pathway analysis in the three horse breeds; Tables S4 and S5: KEGG pathway analysis and GO pathway analysis in the Debao horse are available as Supplementary Materials.
