# Supplementary material for: Genome-Wide Detection of Copy Number Variations Associated with Miniature Features in Horses
Source: Genes (Basel). 2023 Oct 13;14(10):1934. doi: 10.3390/genes14101934 (PMC10606273; doi:10.3390/genes14101934)
Supplement: Supplementary file 1 [file genes-14-01934-s001.zip › Table S1 Summary of samples sequenced in study.pdf]

Table: S1. Summary of samples sequenced in study

Sample name	Number of sequences	Number of bases (bp)	Average sequence length (bp)	Sequencing depth	Alignment percentage (%)
BS01	180643250	27036327450	149.7	10.81453098	99.94
BS02	172455410	25805248216	149.6	10.32209929	99.93
BS03	179578456	26879437920	149.7	10.75177517	99.94
BS04	213752930	31987464124	149.6	12.79498565	99.94
BS05	172264042	25778136512	149.6	10.3112546	99.92
BS06	179096582	26802573276	149.7	10.72102931	99.94
BS07	178730404	26744690486	149.6	10.69787619	99.94
BS08	178030710	26637805940	149.6	10.65512238	99.93
BS09	185967828	27830536868	149.7	11.13221475	99.93
BS10	170798332	25560504490	149.7	10.2242018	99.94
DB01	183125428	27407461344	149.7	10.96298454	99.93
DB02	186377810	27891923450	149.7	11.15676938	99.94
DB03	181638308	27185743512	149.7	10.8742974	99.94
DB04	177754976	26602566854	149.7	10.64102674	99.97
DB05	183032800	27393885818	149.7	10.95755433	99.94
DB06	175456020	26262025676	149.7	10.50481027	99.94
DB07	169420658	25351701650	149.6	10.14068066	99.94
DB08	177286294	26524910610	149.6	10.60996424	99.94
DB09	173516106	25965321682	149.6	10.38612867	99.94
DB10	171389228	25648714332	149.7	10.25948573	99.93
WB01	175413460	26254488820	149.7	10.50179553	99.95
WB02	218837182	32749662596	149.7	13.09986504	99.94
WB03	183595794	27482861162	149.7	10.99314446	99.94
WB04	179200698	26814795436	149.6	10.72591817	99.93
WB05	188065484	28150993900	149.7	11.26039756	99.94
WB06	185837636	27816279662	149.7	11.12651186	99.95
Average	181587147.2	27175617761	149.67	10.8702471	99.94
