# Supplementary material for: Genome-Wide Detection of Copy Number Variations Associated with Miniature Features in Horses
Source: Genes (Basel). 2023 Oct 13;14(10):1934. doi: 10.3390/genes14101934 (PMC10606273; doi:10.3390/genes14101934)
Supplement: Supplementary file 1 [file genes-14-01934-s001.zip › Table S2 KEGG pathway analysis and Tabel S3 GO pathway analysis in three horse breed.pdf]

Functional Annotation of CNVR in three horse breed

Table S2 KEGG pathway analysis

KEGG Pathway	Gene Count	P Value	Gene
ecb04612:Antigen processing and presentation	14	1.41E-04	MHCX1、KLRD1、HSPA4、HSPA1L、IFNG、CTSB、LRRC9、EQMCE1、DRB1、DQA3、DQB1、DQA1、DRB2、DQA2
ecb05321:Inflammatory bowel disease	12	3.89E-04	DRB1、IFNGR2、IL12RB2、IFNG、FRAS1、FRAS1、IL23R、DQB2、DQA1、DRB2、IL18RAP、DQA2
ecb04726:Serotonergic synapse	16	4.14E-04	ALOX12、HTR1E、ALOX5、GNAI2、KCNJ6、HTR7、HTR1A、CYP2C108、CYP4X1、HTR5A、GNAO1、PLA2G4A、HTR1F、KCND2、GNG11、ALOX15B
ecb05145:Toxoplasmosis	15	6.58E-04	MAP3K7、IFNGR2、CCR5、HSPA1L、ALOX5、IFNG、GNAI2、DQB2、DRB1、GNAO1、DQA3、DQB1、DQA1、DRB2、DQA2
ecb05150:Staphylococcus aureus infection	14	0.001589502	KRT10A、DEFA31L、DQB2、DEFA22、DEFA5L、DRB1、DQA3、DQB1、CFH、DQA1、DRB2、ITGB2、ECATH-3、DQA2
ecb05323:Rheumatoid arthritis	13	0.001979771	IFNG、DQB2、DRB1、DQA3、DQB1、CXCL6、ATP6V1H、CXCL8、DQA1、ACP5、DRB2、ITGB2、DQA2
ecb04514:Cell adhesion molecules	18	0.002371425	F11R、MHCX1、CDH2、CNTN1、VCAN、DQB2、VTCN1、PTPRC、EQMCE1、DRB1、ITGA8、DQA3、DQB1、ITGB8、DQA1、DRB2、ITGB2、DQA2
ecb05332:Graft-versus-host disease	11	0.002491976	EQMCE1、DRB1、MHCX1、KLRD1、IFNG、DQA3、DQB1、DQB2、DQA1、DRB2、DQA2
ecb05140:Leishmaniasis	11	0.004276101	MAP3K7、DRB1、IFNGR2、IFNG、FRAS1、

			FRAS1、LRRC9、DQA1、FRAS1、ITGB2、DQA2
ecb05330:Allograft rejection	10	0.004827152	EQMCE1、DRB1、MHCX1、IFNG、FRAS1、FRAS1、LRRC9、DQA1、FRAS1、DQA2
ecb04940:Type I diabetes mellitus	10	0.010278294	EQMCE1、DRB1、MHCX1、IFNG、FRAS1、FRAS1、LRRC9、DQA1、FRAS1、DQA2
ecb05416:Viral myocarditis	10	0.010278294	EQMCE1、DRB1、MHCX1、DQA3、DQB1、DQB2、DQA1、DRB2、ITGB2、DQA2
ecb04080:Neuroactive ligand-receptor interaction	28	0.010966373	UCN3、HTR1E、NPY、OPRM1、ADCYAP1、PTH2R、TACR3、PTGER3、DRD1、HTR5A、GABRG1、THRB、SST、HTR1F、CYSLTR2、EDN3、NMBR、GLRA3、HTR7、SSTR1、HTR1A、TRHR、GHR、QRFPR、GRID2、P2RX6、GABRR1、CHRM3
ecb04742:Taste transduction	10	0.014830627	SV2C、HTR1E、TAS1R2、TAS2R40、HTR1F、TAS2R14、PKD2L1、HTR1A、TAS2R42、CHRM3
ecb05320:Autoimmune thyroid disease	10	0.017015378	EQMCE1、DRB1、MHCX1、IFN-ALPHA2、FRAS1、FRAS1、LRRC9、DQA1、FRAS1、DQA2
ecb04921:Oxytocin signaling pathway	14	0.01768501	PRKAB2、CAMK2D、GNAI2、KCNJ6、EGFR、RGS2、GNAO1、PLA2G4A、MAP2K2、ROCK2、RYR2、CACNB2、ROCK1、TRPM2
ecb04640:Hematopoietic cell lineage	12	0.019640595	DRB1、CD1B2、DQA3、DQB1、CD1B1、DQB2、CD1E1、GYPA、DQA1、DRB2、TFRC、DQA2
ecb05166:Human T-cell leukemia virus 1 infection	19	0.026746256	MHCX1、MSX2、MAP3K1、ETS1、DQB2、EQMCE1、DRB1、MAP2K4、DQA3、DQB1、MAP2K2、DQA1、ANAPC5、BUB3、CCNE1、DRB2、ITGB2、DQA2、SLC25A4
ecb04658:Th1 and Th2 cell differentiation	10	0.028135098	DRB1、IFNGR2、IL12RB2、IFNG、FRAS1、FRAS1、LRRC9、DQA1、FRAS1、DQA2
ecb05152:Tuberculosis	16	0.028156201	CAMK2D、IFNGR2、KSR1、IFN-ALPHA2、IFNG、DQB2、DRB1、PIK3C3、DQA3、DQB1、ATP6V1H、DQA1、DRB2、ITGB2、ECATH-3、

			DQA2
ecb04530:Tight junction	15	0.03200504	PRKAB2、F11R、HSPA4、CTTN、CD1B2、MAP3K1、RAPGEF2、MICALL2、NEDD4、CD1B1、ACTR3B、ROCK2、CD1E1、TJP1、ROCK1
ecb04024:cAMP signaling pathway	17	0.044243718	CAMK2D、EDN3、HTR1E、GLI3、GNAI2、NPY、SSTR1、HTR1A、ADCYAP1、PTGER3、DRD1、SST、MAP2K2、HTR1F、ROCK2、RYR2、ROCK1
ecb01100:Metabolic pathways	86	0.048244214	GALNTL5、SGMS1、NANP、CARNMT1、ENO4、SELENBP1、PLOD2、ALOX5、MINPP1、POMK、B3GALT1、ADPGK、EHMT1、PLA2G4A、ATP6V1H、CYP3A96、TYRP1、CYP7A1、GALNT14、ST6GAL2、PLCz、AACS、NDST4、BCKDHB、SI、ST6GALNAC4、MOCS1、CYP11B1、HYAL4、EXTL3、KYNU、MGAT4C、LPCAT4、PIGB、PIP5KL1、GUCY2D、LBR、DSE、ALOX15B、MAN1A2、B3GALNT1、BLVRA、ADK、FAH、GCNT1、PLCZ1、CYP2B46、CNDP2、PIK3C3、ENPP3 、PRXL2B、PDE5A、CNDP1、GSDMC、CHST10、FGGY、SORD、MAT2B、MTCL1、NT5E、ACP5、ACADSB、SGPL1、ALOX12、ACO1、ALG14、NAMPT、ENPP3、INPP4B、SEMA3E、ELOVL5、GNPDA2、C1GALT1、ABO、GLDC、CYP2C108、SPTLC3、SPAM1、GSTA3、CYP3A89、ENPP1、RRP1、ND5、GSTA1、UXS1、LTA4H

Table S3 GO pathway analysis

Category	Term	Gene Count	P Value	Gene
GOTERM_BP_DIRECT	GO:0019882~antigen processing and presentation	10	2.39E-05	EQMCE1、DRB1、MHCX1、DQA3、DQB1、RAB4A、LRRC9、DQA1、FRAS1、DQA2
GOTERM_BP_DIRECT	GO:0007268~chemical synaptic transmission	15	0.00368292	HTR1E、SNCA、GLRA3、HTR7、HTR1A、OR5T40、HTR5A、GABRG1、LRP6、SV2C、SDCBP、HTR1F、CACNB2、GABRR1、CHRM3
GOTERM_BP_DIRECT	GO:0000902~cell morphogenesis	10	0.005961947	CDH8、CDH7、YAP1、CYFIP1、CDH12、CDH19、CDH11、ARHGEF28、AP3B1、EGFR
GOTERM_BP_DIRECT	GO:0043066~negative regulation of apoptotic process	19	0.024972288	GLI3、BCL2A1、DAPK1、PREX1、PRAME、VTCN1、ZFAND6、CITED2、EGFR、HIPK3、RASA1、SPRY2、CDC73、PSEN1、TCL1A、WNT7A、CAPN13、TFRC、ASCL1
GOTERM_BP_DIRECT	GO:0035556~intracellular signal transduction	24	0.041473254	CLNK、STK32A、PLCZ、PREX1、CAB39L、ASB18、DAPK1、ARHGEF28、TNS3、PLCZ1、SRPK1、STK39、DCDC2C、MARK2、RGS6、PSEN1、SGK1、DCDC2、SDCBP、RPS6KA1、SPSB4、CXCL8、GUCY2D、TFRC
GOTERM_BP_DIRECT	GO:0007156~homophilic cell adhesion via plasma membrane adhesion molecules	11	0.046559993	CDH8、CDH7、PCDH18、CDH2、CDH12、CDH19、KIRREL3、PCDH20、CDH11、CELSR3、PCDH10
GOTERM_CC_DIRECT	GO:0005887~integral component of plasma membrane	55	0.00307342	SGMS1、IFNGR2、SLC26A8、SYNDIG1、PCDH20、OPRM1、SLC39A12、GABRG1、HTR1F、CYSLTR2、SEMA6D、SLC28A2、PCDH10、NMBR、TMPRSS11A、HTR7、HTR1A、CEMIP2、PROKR1、DCBLD2、KIRREL3、QRFPR、TRPM1、IYD、CHRM3、OR56A1、OR4E2、KLR

				D1、HTR1E、MERTK、GYPA、OR5T40、DRD1、HTR5A、PSEN1、SEMA3E、ABCA1、ALK、PCDH18、SLC26A11、SLCO1B3、ADGRL3、GLRA3、SSTR1、ABCB5、KCNK1、GHR、SLC26A7、PKD2L1、SLCO4C1、KCNK2、P2RX6、TFRC、TRPM2、GABRR1
GOTERM_CC_DIRECT	GO:0045202~synapse	23	0.009198062	CYFIP1、HTR1E、RIMS2、SNCA、GNAI2、GLRA3、HTR7、HTR1A、AP3B1、CBLN4、OR5T40、HTR5A、PSEN1、CBLN2、GABRG1、LRP6、SDCBP、HTR1F、LY6E、ACTBL2、CACNB2、GABRR1、CHRM3
GOTERM_CC_DIRECT	GO:0005737~cytoplasm	159	0.030272449	PRKAB2、TRIM45、SMAP1、ALOX5、AMPH、PPP1R14C、PRUNE2、MAPKAP1、PDE4DIP、CSRP1、ELP4、DUSP10、PRMT3、ACTBL2、PPFIA1、LYPLA1、TACC3、CAPN2、TTK、CARS1、SASH1、IRAK1BP1、PRAME、TDRD7、ASPM、HOOK1、SRPK1、TNFAIP3、VWA8、HIPK3、MOB1B、SKP1、MARCHF1、OAS1、ARRDC1、KLHL25、TNK2、VRK2、ROPN1L、FBXO4、NIFK、SIPA1L1、SNCA、MERTK、SPHKAP、CXXC4、ARHGEF11、NOVA1、PIK3C3、AGA、MAP2K2、CDIN1、SERPINB8、BTG1、ENAH、ZNF804A、CYFIP1、HTATIP2、MAP3K1、SERPINB5、DAPK1、WDPCP、DZIP3、CHD1、NUDCD1、PLK2、PTPN22、UXS1、ATXN7L3B、ARHGAP28、KCNIP4、KSR1、UFM1、PADI4、CDH2、UBASH3B、PRPF19、BATF、ACVR2A、RWDD3、MYO5B、URI1、PLA2G4A、ROCK2、ROCK1、DYRK2、PADI2、CAMK2D、UBR3、TBCA、GLI3、NOX3、KIF15、TENT5A、KIZ、TRIM68、NEK1、RGS2、KYN

				U、NEDD4、DOK5、SGK1、GARNL3、SDCBP、CDH11、PARP14、CCNE1、ALOX15B、DEUP1、MRTO4、RNF167、NAP1L4、CAPN8、BLVRA、TRIM21、TRIM26、STXBP5、ANXA10、CYP2B46、FTO、ARRDC4、MARK2、SAMD5、ELP3、CFAP221、LGALS3、SNTG1、CAPN13、YAP1、MAP3K7、ACO1、ASAP2、HSPA1L、NAMPT、CAPN9、ENC1、INPP4B、NR5A2、ARHGEF28、GNPDA2、PADI3、CYP2C108、SCGB1A1、PTPN11、RBFOX2、PMP2、ANXA5、PCMTD1、CPSF6、LRRCC1、DUSP22、FKBP1A、LRRC9、RAP1GAP2、PLAAT1、PARP15、CDADC1、PSPC1、LTA4H
				TMX3、CDH2、GPC5、ACVR2A、HJV、RTP2、TFPI、SLC7A11、EGFR、ITGA8、HYAL4、LRFN2、TSPEAR、PSEN1、LRFN5、ADAMTS15、DCBLD2、LRP6、ENPP1、WNT7A、ITGB8、PKD2L1、SULF2、ZPLD1
GOTERM_CC_DIRECT	GO:0009986~cell surface	24	0.041485424	TMX3、CDH2、GPC5、ACVR2A、HJV、RTP2、TFPI、SLC7A11、EGFR、ITGA8、HYAL4、LRFN2、TSPEAR、PSEN1、LRFN5、ADAMTS15、DCBLD2、LRP6、ENPP1、WNT7A、ITGB8、PKD2L1、SULF2、ZPLD1
GOTERM_MF_DIRECT	GO:0030594~neurotransmitter receptor activity	10	0.007542982	OR5T40、HTR5A、HTR1E、GABRG1、GLRA3、HTR1F、HTR7、HTR1A、GABRR1、CHRM3
GOTERM_MF_DIRECT	GO:0005509~calcium ion binding	41	0.011430849	KCNIP4、CAPN8、EYS、MAN1A2、CDH2、PADI4、VCAN、CDH12、PCDH20、ANXA10、PLCZ1、CDH8、ENPP3 、PLA2G4A、THBS4、CDH19、EFCAB3、RYR2、CAPN13、SULF2、PCDH10、PADI2、VLDLR、PCDH18、PLCz、SPOC

				K3、CAPN9、CAPN2、CETN1、MCTP2、PADI3、CELSR3、CDH7、CEMIP2、ANXA5、CRNN、MATN1、CDH11、PKD2L1、TRPM2、ALOX15B
GOTERM_MF_DIRECT	GO:0004712~protein serine/threonine/tyrosine kinase activity	19	0.017675014	MAP3K7、CAMK2D、TTK、MERTK、DAPK1、STK39、EGFR、PLK2、MARK2、SGK1、RPS6KA1、EPHA6、TNK2、ALK、ROCK2、FRK、MAP3K21、ROCK1、DYRK2
GOTERM_MF_DIRECT	GO:0004672~protein kinase activity	15	0.045718707	MYO3B、CAMK2D、KSR1、MAP3K1、NEK1、BUB1、SRPK1、POMK、HIPK3、PIK3C3、TRIB2、MAP2K4、TEX14、MAP3K21、GUCY2D
GOTERM_MF_DIRECT	GO:0020037~heme binding	13	0.047036578	PXDNL、EPX、THAP4、CYP11B1、CYP2C108、CYP2B46、CYP4X1、CYP3A89、CYP3A96、CYP4F124、CYP7A1、CYB5R4、FLVCR2
GOTERM_MF_DIRECT	GO:0004674~protein serine/threonine kinase activity	21	0.047891973	STK32A、OBSCN、TTK、DAPK1、ACVR2A、MAP3K1、NEK1、SRPK1、STK39、HIPK3、PLK2、MARK2、MAP2K4、PSKH2、SGK1、RPS6KA1、MAP2K2、ROCK2、VRK2、ROCK1、DYRK2
