# Supplementary material for: Genome-Wide Detection of Copy Number Variations Associated with Miniature Features in Horses
Source: Genes (Basel). 2023 Oct 13;14(10):1934. doi: 10.3390/genes14101934 (PMC10606273; doi:10.3390/genes14101934)
Supplement: Supplementary file 1 [file genes-14-01934-s001.zip › Table S4 KEGG pathway analysis of DB and Table S5 GO pathway analysis of DB.pdf]

Functional Annotation of CNVR in Debao Horse

Table S4 KEGG pathway analysis of DB

Category	Term	Count	PValue	Genes
KEGG_PATHWAY	ecb05150:Staphylococcus aureus infection	13	0.001646253	KRT10A、DEFA31L、DQB2、DEFA22、DEFA5L、DQA3、DQB1、CFH、DQA1、DRB、ITGB2、ECATH-3、DQA2
KEGG_PATHWAY	ecb04514:Cell adhesion molecules	17	0.001647925	F11R、MHCX1、CDH2、CNTN1、CDH5、DQB2、VTCN1、PTPRC、EQMCE1、ITGA8、DQA3、DQB1、ITGB8、DQA1、DRB、ITGB2、DQA2
KEGG_PATHWAY	ecb04612:Antigen processing and presentation	11	0.00243409	EQMCE1、MHCX1、KLRD1、Hsp70、DQA3、DQB1、CTSB、DQB2、DQA1、DRB、DQA2
KEGG_PATHWAY	ecb04024:cAMP signaling pathway	19	0.003343294	CAMK2D、EDN3、PDE3A、HTR1E、GLI3、GNAI2、CNGB3、NPY、SSTR1、HTR1A、SOX9、ADCYAP1、GCG、PTGER3、SST、MAP2K2、HTR1F、ROCK2、ROCK1
KEGG_PATHWAY	ecb04726:Serotonergic synapse	13	0.003576006	HTR1E、ALOX5、GNAI2、HTR7、HTR1A、CYP2C108、CYP4X1、HTR5A、GNAO1、PLA2G4A、HTR1F、GNG11、ALOX15B
KEGG_PATHWAY	ecb05416:Viral myocarditis	10	0.004630848	EQMCE1、MHCX1、DQA3、DQB1、DQB2、DQA1、DRB、ITGB2、CASP8、DQA2
KEGG_PATHWAY	ecb05145:Toxoplasmosis	12	0.00606547	MAP3K7、CCR5、GNAO1、DQA3、ALOX5、DQB1、GNAI2、DQB2、DQA1、DRB、CASP8、DQA2
KEGG_PATHWAY	ecb05323:Rheumatoid arthritis	11	0.006974685	CXCL6、ATP6V1H、DQA3、DQB1、DQB2、ACP5、CXCL8、DQA1、DRB、ITGB2、DQA2
KEGG_PATHWAY	ecb04080:Neuroactive ligand-receptor interaction	25	0.014756543	UCN3、HTR1E、NPY、OPRM1、GALR1、ADCYAP1、PTH2R、GCG、TACR3、PTGER3、HTR5A、GABRG1、SST、HTR1F、CYSLTR2、EDN3、NMBR、HTR7、SSTR1、HTR1A、GHR、QRFPR、GRID2、GABRR1、CHRM3
KEGG_PA	ecb04921:Oxy	13	0.0161686	PRKAB2、CAMK2D、GNAI2、EGFR、

THWAY	tocin signaling pathway		08	MYLK3、RGS2、GNAO1、PLA2G4A、MAP2K2、ROCK2、CACNB2、ROCK1、TRPM2
KEGG_PATHWAY	ecb01100:Metabolic pathways	78	0.033640681	SGMS1、NANP、CARNMT1、AHCYL2、ENO4、SELENBP1、PLOD2、ALOX5、MINPP1、POMK、B3GALT1、ADPGK、EHMT1、PLA2G4A、ATP6V1H、CYP3A96、TYRP1、CYP7A1、GALNT14、ST6GAL2、PLCD4、NDST4、ASRGL1、SI、ST6GALNAC4、KMT2C、MOCS1、CYP11B1、HYAL4、MGAT4C、LPCAT4、PIGB、HTR5A、TMEM189、GUCY2D、MYLK3、ALOX15B、CYP2B6、MAN1A2、B3GALNT1、BLVRA、ADK、ELOVL3、FAH、FBP2、GCNT1、FBP1、PLCZ1、CYP2B46、CNDP2、PIK3C3、HTR1A、PDE5A、CNDP1、ITGB2、SORD、MAT2B、IMPA1、NT5E、ACP5、ACADSB、SGPL1、ACO1、PDE3A、AK6、NAMPT、ENPP3、CXCL6、GNPDA2、C1GALT1、ABO、CYP2C108、SPTLC3、SPAM1、GSTA3、PIP5KL1、ND5、UXS1

Table S5 GO pathway analysis of DB

Category	Term	Count	PValue	Genes
GOTERM_BP_DIRECT	GO:0007268~chemical synaptic transmission	16	4.05E-04	HTR1E、SYT10、SNCA、HTR7、HTR1A、OR5T40、HTR5A、LRP8、GABRG1、LRP6、SV2C、SDCBP、HTR1F、CACNB2、GABRR1、CHRM3
GOTERM_BP_DIRECT	GO:1990830~cellular response to leukemia inhibitory factor	10	0.005812596	SPRY2、TEX14、PAX6、NUP35、NR5A2、SSTR1、MRPL15、TFRC、EEF1E1、PADI2
GOTERM_BP_DIRECT	GO:0043066~negative regulation of apoptotic process	19	0.008376292	GLI3、DAPK1、NUP35、PRAME、VTCN1、CITED2、EGFR、AMIGO2、GCG、RASA1、SPRY2、CDC73、TCL1A、PROP1、WNT7A、SMO、CAPN13、TFRC、ASCL1
GOTERM_BP_DIRECT	GO:0007156~homophilic	12	0.009196152	CDH2、CDH5、CDH12、FAT3、CDH19、KIRREL3、PCDH20、CDH11、PCDH7、PCDHAC2、CELSR3、

	cell adhesion via plasma membrane adhesion molecules			PCDH10
GOTERM_BP_DIRECT	GO:0010628~positive regulation of gene expression	20	0.028953168	YAP1、ZNF804A、CNTN1、CDH5、ETS1、NGF、SOX9、CITED2、SOX11、SPRY2、PIP、PAX6、WNT7A、FAM98A、ITGB8、SHH、ROCK2、CXCL8、SMO、ROCK1
GOTERM_CC_DIRECT	GO:0045202~synapse	27	8.04E-05	HTR1E、SNCA、GNAI2、SLC16A1、AP3B1、TIAM2、OR5T40、HTR5A、CBLN2、GABRG1、LRP6、HTR1F、ACTBL2、CACNB2、VWC2、CYFIP1、SYT10、RIMS2、CAPRIN1、HTR7、HTR1A、CBLN4、LRP8、SDCBP、LY6E、GABRR1、CHRM3
GOTERM_CC_DIRECT	GO:0005887~integral component of plasma membrane	46	0.020334015	OR56A1、SGMS1、SLC26A8、HTR1E、OR4E2、KLRD1、SYNDIG1、PCDH20、MERTK、OPRM1、PCDH7、GALR1、GYPA、SLC16A1、SLC39A12、OR5T40、HTR5A、GABRG1、HTR1F、CYSLTR2、SEMA6D、PCDH10、ADGRE1、SLC26A11、SLCO1B3、NMBR、TMPRSS11A、HTR7、SSTR1、ABCB5、HTR1A、CEMIP2、PROKR1、KIRREL3、GHR、QRFPR、TRPM1、PKD2L1、PCDHAC2、IYD、SLCO4C1、KCNK2、TFRC、TRPM2、GABRR1、CHRM3
GOTERM_CC_DIRECT	GO:0030425~dendrite	16	0.022636545	HTR1E、KIF21B、GNAI2、HTR7、ZWINT、NGF、HTR1A、OR5T40、HTR5A、MARK2、STX3、GNAO1、FAT3、HTR1F、SMO、CHRM3
GOTERM_CC_DIRECT	GO:0031965~nuclear membrane	11	0.022881059	MYO6、LYPLA1、MINDY3、CDH5、TMEM18、ALOX5、SDCBP、NUP35、RAP1GAP2、CUEDC2、THAP7
GOTERM_CC_DIRECT	GO:0005886~plasma membrane	142	0.025219468	CCR5、CDH12、RAB4A、OPRM1、AMPH、XKR5、MAPKAP1、LRP6、MINDY3、NUP35、TCAF2、SHH、OR11H5、SULF2、SEMA6D、PCDH10、ARL4C、LYPLA1、SYT10、PREX1、HTR7、OR10Q2、SRPK1、COBL、CEMIP2、LRP8、PRAME、FAT3、SLC34A2、ARRDC1、OR6V1、LY6E、TNK2、GPD1L、ZP4、OR6X1、RHBDL2、MPP7、GSDMC、CTTN、FRMD8、FBP1、HJV、SOX11、PTH2R、TACR3、RASA1、CLEC12A、NIPA2、PTGER3、LY49B、STX3、GFRA2、SLC5A4、OR5C1、ENAH、LANCL2、ZNF804A、DAPK1、CYFIP1、WDPCP、CELSR3、GAS1、SPAM1、LINGO2、OR6C315、OR11A1、GHR、TFRC、TRPM2、OR5P95、VEPH1、OR4F14Q、OR10C1、F11R、STXBP6、ACVR2A、STEAP1、LAIR1、ATP23、ANO5、CDH19、DYNAP、ROCK2、ROCK1、PALS2、ADGRE1、VANGL1、CST3、OR4F80C、ENOX1、RGS2、MICALL2、

				NEDD4、OR10V1、SDCBP、LMBRD1、CDH11、PARP14、PCDHAC2、OR11A1B、GRID2、GUCY2D、ALOX15B、ADK、CNTN1、PLCL1、PANX1、OR4C199、STXBP5、FTO、OR4P7、CSMD3、LY49F、OR2J3H、TCAF1、LY49E、NT5E、VLDLR、MAP3K7、CYP2B46、OR4F80、SLCO1B3、CAPN9、TAS1R2、DMBT1、ADGRA3、OR2W6、OR5J1、OR4F80B、KCNT2、TMEFF2、PKP2、OR2BD2、DUSP22、SLCO4C1、KCNK2、RPSA、SMO、RGS13、RAP1GAP2、STX17
GOTERM_CC_DIRECT	GO:0005737~cytoplasm	144	0.025721383	PRKAB2、TRIM45、SMAP1、ALOX5、RMDN1、TWIST2、SCAF8、AMPH、PPP1R14C、PRUNE2、MAPKAP1、GCG、PDE4DIP、CSRP1、ELP4、PRMT3、ACTBL2、PPFIA1、LYPLA1、TACC3、CARS1、SASH1、IRAK1BP1、PRAME、ASPM、SRPK1、MYLK3、TNFAIP3、VWA8、MOB1B、SKP1、ARRDC1、KLHL25、TNK2、ROPN1L、FBXO4、SNCA、MERTK、CXXC4、FBP1、ARHGEF11、TIAM2、EYA4、CASP8、NOVA1、PIK3C3、AGA、MAP2K2、CDIN1、ENAH、ZNF804A、CYFIP1、HTATIP2、SERPINB5、DAPK1、WDPCP、CAPRIN1、DICER1、CHD1、PLK2、PTPN22、CEBPB、UXS1、ATXN7L3B、ARHGAP28、KCNIP4、PADI4、CDH2、BATF、ACVR2A、MYO5B、URI1、PLA2G4A、RNF111、ROCK2、ROCK1、PADI2、CAMK2D、SHROOM3、UBR3、TBCA、ASRGL1、GLI3、TENT5A、TRIM68、RGS2、NEDD4、DOK5、SGK1、GARNL3、SDCBP、CDH11、ACTR1B、HSPB8、PARP14、CCNE1、PCDHAC2、ALOX15B、DEUP1、CYP2B6、MRTO4、RNF167、NAP1L4、BLVRA、TRIM21、CRACR2A、FBP2、TRIM26、STXBP5、ANXA10、CYP2B46、FTO、MARK2、SAMD5、POC5、ELP3、FAP221、LGALS3、SNTG1、CAPN13、YAP1、MAP3K7、ACO1、AK6、ASAP2、NAMPT、CAPN9、NR5A2、CLMN、GNPDA2、PADI3、CYP2C108、SCGB1A1、PTPN11、RBFOX2、PKP2、ANXA5、LRRCC1、DUSP22、FKBP1A、LRRC3、PER2、RAP1GAP2、PARP15
GOTERM_CC_DIRECT	GO:0043235~receptor complex	13	0.032987078	VLDLR、NR3C2、MERTK、ACVR2A、IL23R、NT5DC3、KCTD8、EGFR、LRP8、IL12RB2、GHR、GFRA2、PKD2L1
GOTERM_MF_DIRECT	GO:0005509~calcium ion binding	38	0.009666486	KCNIP4、MAN1A2、CDH2、PADI4、CDH5、CDH12、CRACR2A、PCDH20、PCDH7、ANXA10、PLCZ1、MINDY3、PLA2G4A、CDH19、SHH、CAPN13、SULF2、PCDH10、PADI2、VLDLR、PLCD4、ADGRE1、SYT10、SPOCK3、CAPN9、CETN1、PADI3、CELSR3、CEMIP2、LRP8、

	ANXA5、FAT3、MATN1、CDH11、PKD2L1、PCDHAC2、TRPM2、ALOX15B
